# Observations Relative to the Use of Belladonna in Painful Disorders of the Head and Face; Illustrated by Many Cases

**Published:** 1818-02

**Authors:** 


*Observations relative to the Use of Belladonna in painful Disorders of the Head and Face; illustrated by many Cases.* By John Bailey, Surgeon. Octavo, pp. 72. London, 1818.

There are few medical men, in any thing like extensive practice, who have not been repeatedly foiled and humiliated in the treatment of those painful nervous affections of the head and face, to which the attention of Mr. Bailey has been directed. The most lovely part of the creation too are the principal sufferers; and the medical attendant being anxiously looked to, both by patient and friends, for relief from pain, his total failure is very apt to impress the spectators with no very exalted ideas of the resources of medicine. From *self interest*, therefore, as well as philanthropy, the profession will joyfully hail any proposal which promises an alleviation of human affliction, and an additional resource in the moment of exigency.

Mr. Bailey comes before the public with little ostentation and much modesty. His work is not expanded by quotations, nor swelled with hypothesis; but contains many important facts, and several judicious observations. As he has rendered the book by its size and price, accessible to every member of the profession, and as we can conscientiously recommend its admission into every medical library, we shall give a less detailed analysis of its contents than the value of the matter may seem to demand.

Mr. B. was led to the employment of Belladonna in neuralgic affections of the face, from having, in 1812, administered a five-grain dose of the extract to a lady, which was attended with darkness of vision, *a benumbed state of the face,* and various other alarming symptoms. He recommends the extract and tincture of the herb as prepared by Corbyn and Co. and details nearly thirty cases of neuralgia facialis, where it was more or less successful.

"Of the tincture, from twenty to forty minims may be given at a dose, in any mild vehicle, augmenting or diminishing it according to its effects, and repeating it with that frequency which the degree of uneasiness which it is intended to subdue requires. Of the extract, prepared agreeably to the directions of the London Pharmacopœia, I at first began with a single grain, and repeated it every four hours, until relief followed; but, upon a further and improved acquaintance, I found it more successful to commence with three times that quantity; and if a repetition were necessary, to give it in diminished doses afterwards." P. 14.

For the reasons stated above, we shall not attempt to abbreviate any of the cases, nor condense the series of judicious comments by which they are accompanied, trusting, as we do, that this meritorious little volume will find its way wherever medical science is zealously cultivated. But we shall make a few remarks on the pathology of this cruel disease; and although our conclusions are somewhat at variance with those of our author, they do not, in any degree, affect the reputation of the remedy he proposes, nor materially clash with his general mode of treatment.

Mr. Bailey considers what has been termed *tic douloureux* as *a local disease,* and having "its origin in the diseased state of membranes lining the cavities of the molar teeth." In short, that the remote cause is a decayed state of the molares, and the immediate or proximate cause, "a diseased state of the extremities of the affected nerves." P. 25.—Now, although we readily admit that the sentient extremities of the nerves are often the seat of the disease, and that their derangement is occasioned by the irritation of carious teeth; yet we are far from thinking that this is *always,* or even generally the case. Mr. B. very justly observes, that neuralgia facialis and hemicrania commonly arise in persons of "irritable habit; for the most part between the 40th and 50th years, and are excited into action by exposure to a cold and humid atmosphere; by fatigue; by external violence; and by uneasiness of mind." Now these circumstances being held in view, we shall have much reason to connect the local *effect* with constitutional *causes*. Without dwelling on the *derangement of function in the digestive organs*, which the above circumstances are calculated to produce, and the local morbid sympathies which so frequently ensue; we may advert to a still more fertile source:—viz. Rheumatic and gouty irritation. When we contemplate how much the climate and modes of life in this country predispose to, and excite the above-mentioned diseases; and when we recollect that the neurilema or envelope of the nervous cords is a structure or tissue which forms the favourite seat of gouty and rheumatic inflammation; and finally, when we bear in mind how much the head and face are exposed to all kinds of atmospherical vicissitudes, we may readily conceive that neuralgic affections must be extremely prevalent.

Nor does even the local treatment or cure of the disease offer any material objection to this view of the subject. Local effects often become, in some measure, independent of their *general* causes, and are cured by topical applications. By paralyzing the facial nerves, we have no doubt that tic douloureux may be cured; at the same time, we think that attention to the general health will be extremely useful, if not necessary, in guarding against relapse. Calomel, opium, and antimonial powder, a combination the most efficacious that has yet been discovered anterior to belladonna, by equalizing the balance of the circulation and excitability, and thus removing the local irritation, elucidate the principle we have now in view.

From attentive observation of the complaint under consideration, for some years past, we are convinced that *tic douloureux* is very frequently an arthritic affection, *en masque*. The violence and periodicity of its attacks, as well as many other of its phænomena, corroborate this opinion.

But such are the obstinacy and intractability of the disease—such the inefficacy of the medicines hitherto employed—and such the misery of disappointment in treating so torturous a malady, that the medical profession will, we are sure, most gratefully receive Mr. Bailey's contribution to therapeutical science, and evince their gratitude by universal perusal of the work.

